# P-1206. Dengue Epidemic Outbreak 2023-2024: is it here for good? Experience in a Tertiary Care Pediatric Hospital in Buenos Aires, Argentina

**DOI:** 10.1093/ofid/ofae631.1388

**Published:** 2025-01-29

**Authors:** Sofia S Carril, Soledad Tineo, Antonella Antonelli Sanz, Agustina Denardi, Andrea Marin, Alicia S Mistchenko, Eduardo L López

**Affiliations:** Hospital de Niños "Dr. Ricardo Gutierrez", CABA, Ciudad Autonoma de Buenos Aires, Argentina; Hospital de Niños Ricardo Gutierrez, Ciudad Autonoma de Buenos Aires, Ciudad Autonoma de Buenos Aires, Argentina; Hospital de Niños "Dr. Ricardo Gutierrez", CABA, Ciudad Autonoma de Buenos Aires, Argentina; Hospital de Niños "Dr. Ricardo Gutierrez", CABA, Ciudad Autonoma de Buenos Aires, Argentina; Hospital de Niños "Dr. Ricardo Gutierrez", CABA, Ciudad Autonoma de Buenos Aires, Argentina; Ricardo Gutierrez Children's Hospital, Buenos Aires, Ciudad Autonoma de Buenos Aires, Argentina; Pediatric Infectious Disease Program, Hospital de Niños Ricardo Gutiérrez, Universidad de Buenos Aires, Ciudad Autónoma de Buenos Aires, Buenos Aires, Argentina

## Abstract

**Background:**

Between epidemiological weeks (EW) 1 and 13 of 2024, the Region of the Americas reported 4,820,955 suspected dengue cases, triple the count from 2023. Argentina ranked second with 379,341 cases, a fourfold increase from the previous season. Our aim is to describe the behavior of dengue in a pediatric population during 2023 and 2024 and compare the characteristics of the latest two dengue outbreaks in Argentina.

**Figure:**
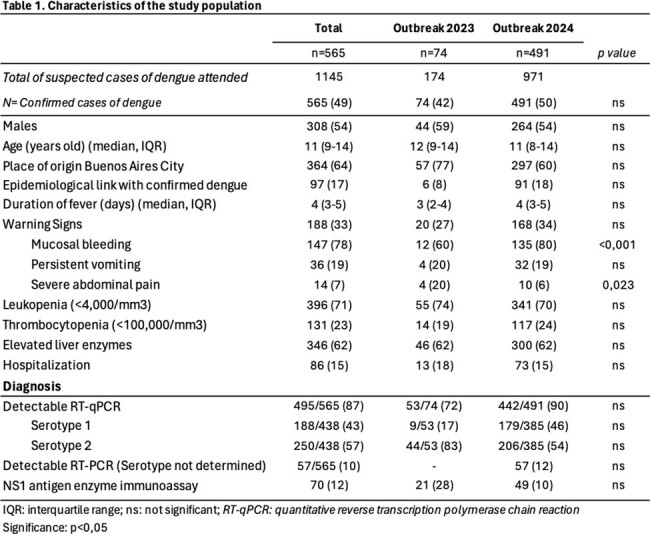

**Methods:**

Two cross-sectional studies were conducted in a children’s hospital in Buenos Aires City. Clinical, epidemiological, laboratory, and follow-up data were recorded for patients < 18 years with confirmed dengue, according to PAHO definitions. The first season period included from March to May 2023 (EW 11 to 20) and the second from January to April 2024 (EW 2 to 15).

**Results:**

A total of 1145 suspected cases were attended. 565 (49%) patients with confirmed dengue were included. Median age: 11 years (IQR 9-14), 308 (54%) being male. Most common symptoms: fever (100%), median duration of 4 days (IQR 3-5); headache/retro-orbital pain (88%); myalgias (75%). 188 patients (33%) exhibited warning signs, the most common: mucosal bleeding, persistent vomiting and severe abdominal pain. 396 patients had leukopenia (71%) and 346 had elevated liver enzymes (62%). Thrombocytopenia (< 100,000/mm^3^) was found in 131 patients (24%). Significant differences were observed when comparing warning signs: more mucosal bleeding in 2024 (p< 0,001), and more abdominal pain in 2023 (p=0,023). Regarding diagnosis, 495 (88%) patients were confirmed by detectable RT-qPCR, serotype 2 predominated (57%) and was significantly most frequent in 2023 (p< 0,001). 70 (12%) patients had positive NS1 antigen enzyme immunoassay. 86 patients (15%) required hospitalization. 5 cases (0,8%) of severe dengue. No deaths occurred.

**Conclusion:**

Timely and precise diagnosis, along with proper categorization of severity criteria, are crucial during dengue infection. One third of patients had warning signs. Despite serotype 2 being the most common during the 2023 outbreak, which is often linked with severe dengue, the hospitalization rate was the same in both periods. The new quadrivalent vaccine available could be an important tool to decrease the number of patients in our region.

**Disclosures:**

**All Authors**: No reported disclosures

